# Vitamin D Intake in a Population-Based Sample of Young Polish Women, Its Major Sources and the Possibility of Meeting the Recommendations

**DOI:** 10.3390/foods9101482

**Published:** 2020-10-17

**Authors:** Zofia Utri, Dominika Głąbska

**Affiliations:** Department of Dietetics, Institute of Human Nutrition, Warsaw University of Life Sciences (SGGW-WULS), 159c Nowoursynowska Street, 02-776 Warsaw, Poland; zofia_utri@sggw.edu.pl

**Keywords:** vitamin D, intake, sources, diet, fish, fish products, species, young women, Poland

## Abstract

The recommendations of vitamin D intake are commonly not met, which results from the fact that fish, being its major sources, are commonly rarely consumed. Consequently, a reliable estimation of its habitual intake is also difficult, as its daily intake is highly variable. The aim of the study was to analyze vitamin D intake from food, its major sources and the possibility to meet its recommendations in a population-based sample of young Polish women. The study was conducted in a sample of Polish women aged 15–30 years, recruited in cooperation with local students’ and youth organizations from all regions of Poland (convenience sampling with the snowball effect), while the stratified sampling procedure was applied with a random quota sampling for voivodeships (an administrative subdivision), to obtain an adequate distribution regarding the general population of young Polish women (*n* = 1,032). The vitamin D intake was assessed while using the validated Vitamin D Estimation Only—Food Frequency Questionnaire (VIDEO-FFQ) and was compared with the recommended 10 µg. The median vitamin D intake in the study group was 3.09 µg (0.00–24.52 µg) and in 95% of participants was lower than recommended, while the highest vitamin D intake was observed for the following sources: eggs (0.50 µg), meat and meat products (0.49 µg), herring, sardine and tuna products (0.41 µg) and dairy products (0.40 µg). The correlation between total vitamin D intake and its intake from its sources was strongest for eggs (*p* < 0.0001; R = 0.5989) and for herring, sardine and tuna products (*p* < 0.0001; R = 0.5314), while the correlation between total vitamin D intake and the number of servings was strongest for herring, sardine and tuna products (*p* < 0.0001; R = 0.5314). At the same time, while compared with other fish species, consuming herring was the strongest predictor of meeting the recommended vitamin D level of 10 µg (*p* = 0.0292; odds ratio (OR) = 1.94; 95% confidence interval (CI) 1.07–3.52), but also of 5 µg (*p* < 0.0001; OR = 2.54; 95% CI 1.85–3.47). Therefore, taking into account the relatively low prices of herring, its high vitamin D content, as well as its influence on total vitamin D intake, it could be beneficial to recommend young women to increase herring intake in order to increase dietary vitamin D intake and to meet its recommendations.

## 1. Introduction

Vitamin D is a micronutrient widely discussed over the years. Its importance especially for the skeletal system is well known, as it promotes calcium and phosphorus absorption in the gut, and maintains their adequate concentrations and bone growth [1]. Nevertheless, while some meta-analyses indicated the effect of its supplementation on reducing the risk of fractures [2] and falling [3], other, more recent ones, suggest that vitamin D supplementation does not prevent either fractures [4,5] or falls and does not have meaningful effects on bone mineral density [5], which shows that there still is a need for further research. However, vitamin D does not influence bone health only [6]. Other meta-analyses show that its high concentrations are found to be associated with a decreased risk of cardiometabolic disorders (cardiovascular disease, type 2 diabetes and metabolic syndrome) among middle-aged and elderly populations [7], breast cancer in premenopausal women [8] and autoimmune thyroid disease among adults in general [9]. At the same time, low vitamin D levels are associated with an increased risk of depression [10], impaired cognitive function [11], Alzheimer’s [11] and Parkinson’s disease [12], attention deficit hyperactivity disorder (ADHD) in children and adolescents [13], and progression of tuberculosis disease [14]. Other studies indicate that vitamin D supplementation might improve vascular function in chronic kidney disease patients [15], while its higher concentration may reduce the risk of fractures in human immunodeficiency virus (HIV) patients [16]. Further meta-analyses also indicate an association between its supplementation and improved biochemical parameters of bone metabolism in chronic kidney disease patients [17], as well as a reduced risk of acute respiratory tract infection [18], colorectal cancer incidence [19], and cancer death [20].

On the other hand, the issue of the recommended vitamin D intake arouses a great deal of controversy. National recommendations for adults vary from 5 µg in Australia and New Zealand [21], 10 µg in the United Kingdom [22] and in the Nordic countries [23], 15 µg in France [24] and in Poland [25] to 20 µg in Germany, Austria and Switzerland [26]. At the same time, the American Institute of Medicine Committee set the Dietary Reference Intakes (DRIs) for vitamin D for adults at 10 µg (estimated average requirement, EAR) and 15 µg (recommended dietary allowance, RDA) [27]. However, the European Food Safety Authority (EFSA) Panel on Dietetic Products, Nutrition and Allergies (NDA) did not formulate the EAR/RDA values for the European population, but instead the adequate intake (AI) value, which was set at 15 µg (when there is minimal cutaneous vitamin D synthesis) and underlined that in the presence of cutaneous vitamin D synthesis the requirement is lower and may even be zero [28].

In spite of the fact that there is no agreement on the recommended vitamin D intake, it is commonly stated that there is a problem of its insufficient intake, which has been observed in many countries worldwide [29,30,31,32]. In Europe, according to the European Prospective Investigation into Cancer and Nutrition (EPIC) study, the mean vitamin D intake in adults aged 35–74 years is 4.8 µg for men and 3.3 µg for women [31], whereas in Poland for men 5.7 µg and for women 3.3 µg [33]. Beside the fact that vitamin D intake is lower in women than in men, while the recommended intakes are the same [21,22,23,24,25,26,27], the prevalence of osteoporosis and osteoporotic fractures is more common among females than males [34]. Among women the problem of inadequate vitamin D intake is most serious amidst young women, as in the group aged 19–30, according to the 2003–2006 National Health and Nutrition Examinations Survey (NHANES), the vitamin D intake is the lowest from all female groups [35]. Moreover, bones reach their peak bone mass and density by the age of 30 and afterwards bones mass loss begins; therefore, until that age it is essential to effectively prevent future bone health problems, such as osteoporosis [36]. That is why young women (until the age of 30) are the population for whom the problem of inadequate vitamin D intake is most serious.

The other issue is the reliable assessment of vitamin D intake, as it is found only in few very specific food products, mainly fish [37,38], which are often not consumed every day—e.g., in France, Italy, Spain, Germany, the United Kingdom and the Netherlands the highest fish consumption is observed on Fridays [39]. As a result, while using the commonly applied short-term dietary intake methods such as the dietary recall or food record, it may be impossible to obtain precise data on habitual vitamin D intake. Such a situation is observed in Poland, as in the studies conducted either a single 24 h dietary recall [40] or a 2-day [41] or 3-day food record [42] methods were used to access vitamin D intake. A similar situation is observed for other countries, as a number of prominent studies were also based on such methods, as for the EPIC study (single 24 h dietary recall) [32] or the latest Finnish population-based study (2 non-consecutive 24-hour dietary recalls) [43]. As a result, it is indicated that for vitamin D, to obtain reliable information, rather the other methods should be applied to assess its habitual intake, as in the Food4Me study, which was conducted in 7 European countries (including Poland) and used a screening food frequency questionnaire (FFQ) [44]. However, to date, only few such studies were conducted and no such study has been conducted among large homogenic groups of Polish respondents.

Despite limited sources of vitamin D, some studies show that total vitamin D intake and status may depend not only on its supply from fish, but also from its minor sources [45]. This indicates that there might be specific food products which may have an influence on vitamin D intake and, in consequence, vitamin D status. This may depend not only on vitamin D content in the product, but also on the frequency of its consumption, which results from the practical possibilities to include it into a habitual diet. Taking it into account, such products should be recognized to be recommended to increase vitamin D intake.

Therefore, the aim of the presented study was to analyze vitamin D intake from dietary sources, its major sources and the possibility to meet its recommendation in a population-based sample of young Polish women, while using a vitamin D-specific FFQ validated for this population. The hypothesis of the presented study was that vitamin D intake from food is associated with the intake of specific food products and that there are some fish species which have the most significant influence on its intake and which could be recommended in order to increase vitamin D intake.

## 2. Materials and Methods

The study was conducted according to the Guidelines of the Declaration of Helsinki. All the participants provided their informed consent. The study procedure was accepted by the Bioethical Commission of the National Food and Nutrition Institute in Warsaw (No. 0701/2015).

### 2.1. Study Group

The presented study was conducted in a group of young women living in all regions of Poland. The inclusion criteria were as follows: women, 15–30 years of age and who provided an informed consent to participate in the study. The exclusion criteria were as follows: currently not living in Poland and any missing or unreliable data. There were no additional inclusion/exclusion criteria associated with health or the followed diet, as the aim was to obtain a various sample, as representative for the general population of young Polish females as possible.

The study group was recruited in two stages of the sampling procedure in order to obtain a population-based sample with an adequate respondents’ distribution within the regions and voivodeships (an administrative subdivision) of Poland. Due to this fact, the recruitment was conducted in cooperation with local students’ organizations and youth organizations from all regions of Poland. Students from the organizations were contacted using social media of their organizations and, after receiving an authorization, a link to access the questionnaire with the information about the study (its aim, purpose, methodology, studied group) was posted in each students’ group separately. All members of the groups had the possibility to fill in the questionnaire. However, after answering the questions about the inclusion criteria (gender, age, living in Poland—while the specific voivodeship was to be indicated), it was verified if they met them and if not, the questionnaire was automatically closed down and they were not able to participate in the study. In that stage of the sampling, the participants were recruited using convenience sampling with the snowball effect. Afterwards, in the second stage of the sampling, a stratified sampling procedure was conducted with a random quota sampling for voivodeships—from all the respondents that had completed the questionnaire, a proportional number of respondents was randomly chosen to obtain the final sample characterized by an adequate distribution regarding the general population of young Polish women. The random quota sampling was conducted for the individuals who had met the inclusion criteria and provided a complete form without any missing or unreliable data.

**Figure 1:**
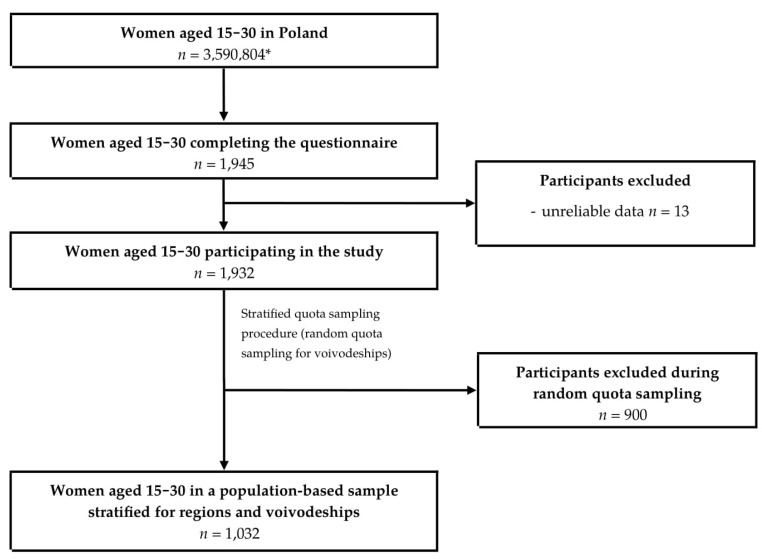
Sampling procedure in the conducted study. * Statistics Poland data for the year 2017 [46].

The study group consisted of 1,032 women aged 15–30 years living in all voivodeships in Poland. The sampling procedure is presented in Figure 1.

### 2.2. Data Gathering

The data were gathered while using the CAWI (computer-assisted web interview) method and the interview was conducted for all respondents willing to participate in the study that have met the inclusion criteria (before the stratified sampling procedure).

In Poland, fish intake may depend on the season, so in order to reduce bias (associated with the fact that fish intake may significantly influence vitamin D supply), the study was conducted in the same period in all the regions of Poland. The period of 11 September–10 November was chosen as a typical time of the year, due to the fact that in Poland an increased fish intake may be observed in July and August (the time of summer holidays, which is frequently spent by many at the seaside), as well as in December (Christmas) and spring months (Lent)—due to the consumption of traditional dishes containing fish.

The developed questionnaire included questions that were necessary to verify the inclusion and exclusion criteria, namely questions about: gender (close-ended single-choice question), age (open-ended question), and living in Poland, while the specific voivodeship was to be indicated (close-ended single-choice question). At the same time, the form to express the informed consent to participate with the personal data protection information was generated and, if confirmed, the main part of the questionnaire was to be completed.

The main part of the questionnaire was based on the brief FFQ to assess vitamin D intake from food that was previously validated in a group of Polish young women (Vitamin D Estimation Only—Food Frequency Questionnaire (VIDEO-FFQ)) [47]. This is a quick and convenient tool of high validity and reproducibility, which was observed for the Polish [47] and Croatian [48] population, which enables assessing vitamin D intake from food in large populations. To date, it has been used to assess vitamin D intake in various studies [49,50] and is included in the Register of Validated Short Dietary Assessment Instruments by the National Institutes of Health (NIH)—National Cancer Institute of United States of America [51].

The applied VIDEO-FFQ questionnaire [47] included questions about the intake of specified food products during the previous year, independent of season, while respondents were asked to specify the number of servings per month, week or day, depending on the product (open-ended questions). The food products within the questionnaire were clustered in groups based on the characteristics of the products and on vitamin D content. For each product the serving size was defined in grams and using typical household measures. Respondents were to specify the amount of products consumed and those added to dishes, while they were allowed to indicate the number of servings not only as integers, but also as decimal parts of servings.

To gather the data the VIDEO-FFQ was applied while using the CAWI method. Respondents had to complete the questionnaire question by question, and therefore to reduce the risk of bias associated with the previously indicated answers, the order of 2 questions was changed while compared with the original form (intake of fresh or smoked fish and of fish products) in order to ask first about the intake of fish products and only then about the intake of fresh and smoked fish. The reason for that was to minimize the risk of overestimation of fresh and smoked fish intake due to the fact that one of the listed fish species there was herring, which is usually eaten in Poland in the form of fish products (such as marinated in vinegar, oil or cream) and not fresh or smoked fish, and could have been mistakenly declared first of all in the fresh and smoked fish question, and then (again) in the fish products questions.

The additional questions, which are included in the VIDEO-FFQ questionnaire [47], about fish species and fish products (out of those listed) which are consumed by the respondents most frequently, were also asked.

Based on the data gathered, the habitual daily vitamin D intake from food was calculated for each respondent while using the formulas developed for the VIDEO-FFQ questionnaire, based on the Polish food composition tables, as described by Głąbska et al. [47], which allow the calculation of the total dietary intake, as well as the intake from specific groups of products, such as: salmon, rainbow trout, herring, eel (group of fish with the highest vitamin D content); halibut, mackerel, brook trout, sole, tuna (group of fish with a medium vitamin D content); cod, flounder, plaice, pollock, hake (group of fish with the lowest vitamin D content); herring, sardine and tuna products (fish products with higher vitamin D content); other fish products (fish products with lower vitamin D content); dairy products; eggs; meat and meat products; cereals and fats.

In order to compare the obtained data to the vitamin D intake recommendations the cut-off point of 10 µg was chosen, as recommended by the American Institute of Medicine Committee as the EAR value [27], by the UK Scientific Advisory Committee on Nutrition [22] and recommended in the Nordic Recommendations [23].

### 2.3. Statistical Analysis

The distribution of the obtained data was verified using the Shapiro–Wilk test. Due to the stated non-parametric distributions, the Spearman rank correlation coefficient was applied to analyze the correlations and the Mann–Whitney *U*-test to compare the sub-groups. The analysis of the logistic regression was conducted while the association between consuming specific fish species and meeting the recommended vitamin D intake for the levels of 5 µg (recommended in Poland while the study was conducted [52]) and 10 µg (recommended by the major international authorities [22,23,27]) was verified. The binomial logistic regression was conducted with the target variable of meeting/not meeting the recommended vitamin D intake.

The accepted level of significance was *p* ≤ 0.05. The correlation coefficients were interpreted while using the classification by Hinkle et al. [53]. The following software was used: Statistica 8.0 (Statsoft Inc., Tulsa, OK, USA), Statgraphics Plus for Windows 4.0 (Statgraphics Technologies Inc., The Plains, VA, USA).

## 3. Results

The study group distributed by regions (6 regions) and voivodeships (16 voivodeships in Poland) is presented in Table 1, while the distribution within regions/voivodeships was verified for its representativeness based on a comparison with the Statistics Poland data. It was indicated that the distribution is in agreement with the general distribution of the Polish population for this age group.

The total vitamin D intake from food and its intake from various food groups in the studied group of young Polish women are presented in Table 2. The median intake of vitamin D in the study group was 3.09 µg (it ranged from 0.00 µg to 24.52 µg) and it was lower than the recommended 10 µg [22,23,27] for 95% of participants. The highest median vitamin D intake from food groups was observed for eggs (0.50 µg, ranging from 0.00 µg to 11.50 µg), meat and meat products (0.49 µg, ranging from 0.00 µg to 5.37 µg), herring, sardine and tuna products (0.41 µg, ranging from 0.00 µg to 12.36 µg) and dairy products (0.40 µg, ranging from 0.00 µg to 4.12 µg), whereas the lowest for the group of fresh or smoked cod, flounder, plaice, pollock and hake (ranging from 0.00 µg to 0.83 µg) and other than herring, sardine and tuna fish products (ranging from 0.00 µg to 0.47 µg).

The declared number of servings of fish and fish products consumed per month in the studied group of young Polish women is presented in Table 3. The median intake of fish species such as salmon, rainbow trout, herring and eel, as well as halibut, mackerel, brook trout, sole and tuna, and herring, sardine and tuna products amounted to 1 serving per month (ranging from 0 to 50 servings for fish and from 0 to 30 for fish products). The lower number of servings was declared for cod, flounder, plaice, pollock and hake, as well as other fish products.

The analysis of correlation between vitamin D intake from its various sources and total dietary vitamin D intake from food in the studied group of young Polish women is presented in Table 4. The analysis revealed a statistically significant association for all sources of vitamin D, but the strongest positive correlation (interpreted as moderate based on the classification by Hinkle et al. [53]) was stated for eggs (*p* < 0.0001; R = 0.5989) and for herring, sardine and tuna products (*p* < 0.0001; R =0.5314).

The analysis of correlation between the declared number of servings of fish and fish products consumed per month and total dietary vitamin D intake from food in the studied group of young Polish women is presented in Table 5. The analysis revealed statistically significant association for all fish and fish products, but the strongest positive correlation (interpreted as moderate based on the classification by Hinkle et al. [53]) was stated for herring, sardine and tuna products (*p* < 0.0001; R = 0.5314).

The analysis of influence of predominantly choosing specific fish species on total vitamin D intake from food, conducted based on the analysis of correlation between vitamin D intake from fish and total dietary vitamin D intake from food, and the comparison of vitamin D intake among participants choosing specific fish species or not, is presented in Table 6. The median total dietary vitamin D intake from food was higher among participants predominantly choosing salmon (3.31 µg, ranging from 0.52 µg to 24.52 µg ) than those not choosing it (2.65 µg, ranging from 0.00 µg to 19.94 µg) (*p* < 0.0001), as well as those predominantly choosing rainbow trout (3.59 µg, ranging from 0.52 µg to 19.37 µg) than those not choosing it (2.90 µg, ranging from 0.00 µg to 24.52) (*p* < 0.0001), and those predominantly choosing herring (3.76 µg, ranging from 0.12 µg to 24.52 µg) than those not choosing it most frequently (2.55 µg, ranging from 0.00 µg to 16.06 µg) (*p* < 0.0001). For sub-groups stratified based on predominantly choosing eel, the median total dietary vitamin D intake did not differ. A stronger correlation (interpreted as moderate and in the case of participants predominantly choosing rainbow trout—high positive, based on the classification by Hinkle et al. [53]) between vitamin D intake from fish and total dietary vitamin D intake from food was observed for participants predominantly choosing salmon (*p* < 0.0001; R = 0.6839 vs. *p* < 0.0001; R = 0.6305), rainbow trout (*p* < 0.0001; R = 0.7157 vs. *p* < 0.0001; R = 0.6603) or herring than for those not choosing those fish species (*p* < 0.0001; R = 0.6791 vs. *p* < 0.0001; R = 0.5870). For eel there was no correlation observed for sub-group predominantly choosing eel (*p* = 0.6514; R = −0.1905), as only a few respondents declared it.

The results of the logistic regression for the association between predominantly choosing specific fish species and meeting the recommended vitamin D intake for the levels of 5 µg and 10 µg are presented in Table 7. A similar analysis was not conducted for meeting the recommended vitamin D intake of 15 µg, as only few respondents (1%) met this recommended level. The analysis revealed that the strongest predictor of meeting the recommended level of vitamin D intake is consuming herring, as it was indicated as a significant predictor both for the 5 µg and 10 µg level of vitamin D intake. For the model developed for meeting the recommended 5 µg level of vitamin D intake, predominantly choosing salmon (odds ratio (OR) = 1.5793; *p* = 0.0050), rainbow trout (OR = 1.5851; *p* = 0.0148) and herring (OR = 2.5356; *p* < 0.0001) increased the possibility of meeting the recommended level, but it was not stated for eel. At the same time, for the model developed for meeting the recommended 10 µg level of vitamin D intake, predominantly choosing herring (OR = 1.9413; *p* = 0.0292) increased the possibility of meeting the recommended level, but it was not stated for the other analyzed species.

## 4. Discussion

In order to formulate applicable dietary recommendations as a way to increase vitamin D intake it is essential to possess actual and accurate data on habitual vitamin D intake and its main sources from research conducted in population samples. The presented study makes this more possible as it is, to date, the first one conducted among a population-based group of young women representing all voivodeships of Poland, in which a validated brief FFQ to assess vitamin D intake [47] was used, which is a much more accurate tool to access vitamin D intake than the frequently used methods such as the 24 h dietary recall or the 3-day food record.

The median vitamin D intake from food in the studied population-based sample of young Polish women was 3.09 µg and it ranged from 0.00 µg to 24.52 µg. Similar vitamin D intakes were observed in a number of studies conducted in Poland in groups of young women. In a study conducted among young women attending Warsaw fitness classes, the median vitamin D intake was 3.18 µg (0.15–34.26 µg) and 77% of the participants did not meet the recommended 10 µg [41]. In another study conducted among young and middle-aged women, the mean vitamin D intake was 3.14 ± 5.22 µg [54], whereas in a Multi-Center National Population Health Examination Survey (WOBASZ Study), the mean vitamin D intake among women of various age was 2.5 ± 2.9 µg [55]. Other, much smaller, studies conducted among female students from various universities, namely the Agricultural University of Szczecin, the Westpomeranian Technological University in Szczecin, the Białystok Institute of Cosmetology and Healthcare and the Rzeszów University revealed an even lower vitamin D intake of 2.2 ± 3.9 µg [56], 2.08 ± 1.68 µg [57], 2.2 ± 2.8 µg [58], and 2.45 ± 2.92 µg, respectively [42]. What should be underlined is that in the majority of studies concluding a lower vitamin D intake than in the presented study, the data were obtained based on a 24 h dietary recall, which is well-known to be a method not of the highest reliability [59]. It is not an accurate method especially for vitamin D intake, due to the fact that vitamin D can be found only in few, very specific food products, and its highest amounts are found in fish [60], which are typically not consumed by Poles every day [39]. Therefore there is a high risk of underestimating vitamin D intake, resulting from a bias associated with recalling dietary intake from a day on which no fish was consumed.

What could be noted is that, while comparing vitamin D intake data from various studies conducted in Poland, vitamin D intake among young women attending fitness classes [41] was higher than in the other studies. What could be presumed in that case is that women attending fitness classes may be more nutrition-cautious and their dietary habits may be more akin to recommendations (as stated that they eat regular meals, consume at least 4 servings of fresh vegetables and fruits every day [41]), which is also observed in other studies [61,62] and they might choose products being sources of vitamin D more frequently than the general population of Polish women.

What should be underlined is that most of the studies mentioned above [41,42,54,56,57,58] are conducted in small, non-homogenous and non-representative samples, while conducted in various periods and regions of Poland. Taking it into account, it is especially valuable to conclude about the possible vitamin D sources to verify the possibility of meeting the recommended intake, based on a large study conducted in a homogenic population-based sample.

The main sources of vitamin D are: skin synthesis of cholecalciferol from 7-dehydrocholesterol [63,64], natural food products containing vitamin D, as well as vitamin D-fortified products and dietary supplements [29]. Vitamin D content in dietary sources varies significantly—it is highest in oily fish (in Poland: such as eel—30 µg/100 g, herring—19 µg/100 g, salmon—13 µg/100 g), much lower in other fish species (such as cod—1 µg/100 g or flounder—0.8 µg/100 g), but can also be found in other animal products (such as meat—1.5 µg/100 g, milk—0.56 µg/100 g, rennet cheese—0.18 µg/100 g; eggs—1.7 µg/100 g) (content based on Polish data) [60].

In the presented study the dietary vitamin D supply was analyzed based on the Polish food composition tables, as described by Głąbska et al. [47]. Interestingly, in the presented study the main habitual dietary vitamin D sources were: eggs (on average 0.50 µg daily), meat and meat products (0.49 µg) and only then fish products (herring, sardine and tuna species) (0.41 µg). At the same time, salmon, rainbow trout, herring and eel, which are fish species of the highest vitamin D content (13–30 µg/100 g), provided on average only 0.26 µg of vitamin D. Such a distribution of vitamin D sources in the diet in the studied population is associated with the fact that the intake of eggs, meat and meat products, as well as milk and dairy products was much higher than for fish and fish products, increasing their role as a source of this nutrient, despite the fact that they contain less vitamin D than fish. The median intake of fish in the study group amounted to 3 servings per month (1 serving of each of the 3 groups of fish). Such a number of servings is a little lower than observed in an international study, in which a semi-quantitative FFQ was used in Central-Eastern European countries, and the intake of fish high in vitamin D was 0.38 servings per day [65]. However, in the referred study, neither the serving size, nor the included fish species were defined [65], so a comparison of the obtained results in the studies is straitened.

The presented study, as well as studies conducted in other countries, indicates the impact of regular fish consumption on a habitual vitamin D intake. The Finnish population-based study FINDIET [66], indicated a general insufficient vitamin D intake in the studied population, while the highest intake was observed for the sub-group characterized by the highest fish consumption. Moreover, according to the FINDIET 2012 study [67]—a more recent study on that population, vitamin D intake was on average sufficient, and its main sources were: fish, dietary fats and milk products fortified with vitamin D. The mean intake of fish dishes among all women from the FINDIET 2012 study (including those not consuming fish at all), was 40 ± 66 g/day, whereas among only those who consumed it 95 ± 72 g/day, while fish dishes provided 2.0 µg (23%) of total vitamin D intake in all participating women [67]. In the presented study, the median vitamin D intake from fish and fish products was 0.73 µg, which contributed to 24% of total vitamin D intake, which may be concluded to be a similar share, but for a lower intake, due to the general lower consumption of fish and vitamin D-fortified products in this study.

In another study, conducted among the Swedish population [68], fish and fish products provided 32% of total dietary vitamin D and, similarly to the previously referred study [67], the highest intake was observed for the sub-group characterized by the highest fish consumption. Simultaneously, in this Swedish study the lowest vitamin D intake (5.2 ± 3.2 µg) was observed for young women, whose fish consumption was the lowest and averaged 24 ± 28 g per day [68]. It confirms the problem concerning this population group, especially since proper vitamin D intake is crucial for bone growth, which occurs until the age of 30 [36]. What should be noted is that even in populations of higher general vitamin D intake, the intake in this population group is often still inadequate, due to the fact that the most effective sources of this nutrient are frequently not included in a diet in sufficient amounts.

A cross-sectional Dutch study [69] confirmed the relationship between fish consumption and vitamin D supply even more clearly and indicated the need for recommending increasing the consumption of fatty fish in order to improve vitamin D status. In the referred study, the strongest correlation with 25-hydroxycholecalciferol blood level was found for the number of fatty fish servings consumed (R = 0.160; *p* < 0.001), so it was concluded that more emphasis should be placed on the consumption of fatty fish as it may effectively improve vitamin D status [69].

The presented study indicated that among the assessed fish species, the consumption of salmon, rainbow trout and herring was most strongly associated with total dietary vitamin D intake from food. This is due to the fact that these fish species contain very high amounts of vitamin D (salmon 13 µg, rainbow trout 13.50 µg, herring 19 µg/100 g [60]) and, while compared with other fish species (such as eel), are relatively frequently consumed. However, at the same time, an even stronger relationship was observed for eggs (which contain 1.70 µg of vitamin D/100 g), being consumed frequently in the study group. For this reason, salmon, rainbow trout and herring are species of fish that could potentially help meet the vitamin D requirements and, therefore, should be recommended in order to effectively increase vitamin D intake in the group of young women.

The analysis of Statistics Poland shows that the general fish consumption in Poland is low, as the average monthly consumption of fish and seafood (excluding fish products) in 2017 was 290 g per person (9.7 g daily) [70]. In a study conducted in a group of young and middle-aged women from the Greater Poland voivodeship [71], it was shown that their average daily fish consumption was 35 g, which corresponds to 245 g per week. The contribution of specific fish species may be analyzed based on the Polish balance sheet data of fish and seafood consumption in 2017 [72], which describes the general fish consumption in Poland as amounted to 12.46 kg/person, i.e., 1.04 kg/person per month, with a high consumption recorded for herring (2.56 kg/year—20.5%), but low for salmon 0.63 kg/year—5.1%, and trout 0.50 kg/year—4.0%. This structure of consumption proves that herring is the most frequently chosen fish species that contains high amounts of vitamin D, which may be due to the relatively low price of herring (16.03 PLN (Polish currency)/kg—approx. 3.63 €/kg), while compared with the higher price of salmon (57.90 PLN/kg—approx. 13.13 €/kg) and rainbow trout (23.62 PLN/kg—approx. 5.27 €/kg) [72]. Moreover, in Poland herring is available on the market in the form of many processed products sold in convenient packaging (e.g., rollmops, herring in cream, “one-bite herring”), so its consumption does not require any preparation.

In the presented study, the origin of the consumed fish was not investigated—participants were not asked whether the fish they choose is bought in supermarkets, fish shops, from a fisherman etc., neither whether it comes from sustainable fishing fisheries and/or holds any fisheries certification such as the Marine Stewardship Council (MSC) Fisheries Standard [73], nor if the consumed fish are from wild or captive-bred feeding regimes. In further research those questions should also be addressed as studies indicate that marine fisheries such as the Baltic Sea are often overexploited [74] and also that the feeding regime (wild or captive-bred) of fish has an influence on its nutritional properties [75].

Taking into account the presented results, in order to effectively increase vitamin D intake from dietary sources, it seems to be crucial to formulate specified recommendations, including the consumption of salmon, rainbow trout and, above all, herring. Some young women already consume it quite often, which may be due to its wide availability (in various forms) and relatively low price. However, the lack of awareness of the nutritional value of herring and the high vitamin D content in it may be why it is not consumed more often. Hence, it might be more beneficial to recommend that young women increase the intake of herring in order to increase dietary vitamin D intake than to recommend increasing fish intake in general, as increasing the intake of this fish species might be more effective in terms of meeting the vitamin D recommendations. At the same time, there is a need for appropriate population-based strategies, including nutritional education, in order to change the commonly observed dietary habits concerning fish consumption into more beneficial ones.

## 5. Conclusions

While compared with other fish species, consuming herring was the strongest predictor of meeting the recommended dietary vitamin D intake. Taking into account the relatively low prices of herring, its high vitamin D content, as well as its influence on total vitamin D intake, it could be beneficial to recommend that young women increase herring intake in order to increase dietary vitamin D intake and to meet recommendations.

## Figures and Tables

**Table 1:** The study group distributed by regions and voivodeships.

Region	Number (%) of Participants	Number (%) of Polish Women Aged 15–30 Living in Each Region *	Voivodeship (within Region)	Number (%) of Participants	Number (%) of Polish Women Aged 15–30 Living in Each Voivodeship *
Central	200 (19.4)	699,529 (19.5)	Łódź	60 (5.8)	219,282 (6.1)
			Masovian	140 (13.6)	480,247 (13.4)
Southern	200 (19.4)	733,949 (20.4)	Lesser Poland	80 (7.8)	335,660 (9.3)
			Silesian	120 (11.6)	398,289 (11.1)
Eastern	192 (18.6)	661,160 (18.4)	Lublin	52 (5.0)	207,901 (5.8)
			Subcarpathian	60 (5.8)	218,962 (6.1)
			Podlaskie	40 (3.9)	116,917 (3.3)
			Holy Cross	40 (3.9)	117,380 (3.3)
North- Western	175 (17.0)	584,007 (16.3)	Lubusz	35 (3.4)	94,611 (2.6)
			Greater Poland	90 (8.7)	333,275 (9.3)
			West Pomeranian	50 (4.8)	156,121 (4.3)
South- Western	100 (9.7)	348,193 (9.7)	Lower Silesian	70 (6.8)	256,906 (7.2)
			Opole	30 (2.9)	91,287 (2.5)
Northern	165 (16.0)	563,966 (15.7)	Kuyavian-Pomeranian	60 (5.8)	200,291 (5.6)
			Pomeranian	65 (6.3)	222,551 (6.2)
			Warmian-Masurian	40 (3.9)	141,124 (3.9)
p **	0.9665		p **	0.7654	

* Based on Statistics Poland data for the year 2017 [46]; ** Verification of representativeness based on a comparison of distributions within regions/voivodeships.

**Table 2:** The total vitamin D intake from food and its intake from various food groups in the studied group of young Polish women.

Vitamin D Source	Mean ± SD (µg)	Median (Min–Max) (µg)
Total intake from food	3.85 ± 2.96	3.09 * (0.00–24.52)
Salmon, rainbow trout, herring, eel	0.51 ± 1.07	0.26 * (0.00–12.75)
Halibut, mackerel, brook trout, sole, tuna	0.10 ± 0.24	0.06 * (0.00–4.17)
Cod, flounder, plaice, pollock, hake	0.02 ± 0.05	0.00 * (0.00–0.83)
Herring, sardine and tuna products	0.59 ± 0.91	0.41 * (0.00–12.36)
Other fish products	0.02 ± 0.04	0.00 * (0.00–0.47)
Dairy products	0.51 ± 0.44	0.40 * (0.00–4.12)
Eggs	0.83 ± 1.04	0.50 * (0.00–11.50)
Meat and meat products	0.64 ± 0.64	0.49 * (0.00–5.37)
Cereals	0.17 ± 0.18	0.13 * (0.00–2.21)
Fats	0.47 ± 1.12	0.09 * (0.00–13.60)

* Non-parametric distribution (verified using Shapiro-Wilk test, *p* ≤ 0.05).

**Table 3:** The declared number of servings of fish and fish products consumed per month in the studied group of young Polish women.

Fish Product Group	Mean ± SD (Servings/Month)	Median (Min–Max) (Servings/Month)
Salmon, rainbow trout, herring, eel	1.97 ± 4.17	1.00 * (0.00–50.00)
Halibut, mackerel, brook trout, sole, tuna	1.19 ± 3.06	1.00 * (0.00–50.00)
Cod, flounder, plaice, pollock, hake	1.19 ± 3.53	0.00 * (0.00–50.00)
Herring, sardine and tuna products	1.43 ± 2.22	1.00 * (0.00–30.00)
Other fish products	0.58 ± 1.19	0.00 * (0.00–15.00)

* Non-parametric distribution (verified using Shapiro–Wilk test, *p* ≤ 0.05).

**Table 4:** The analysis of correlation between vitamin D intake from its various sources and total dietary vitamin D intake from food in the studied group of young Polish women.

Vitamin D Source	*p*	R
Salmon, rainbow trout, herring, eel	<0.0001	0.4914
Halibut, mackerel, brook trout, sole, tuna	<0.0001	0.3641
Cod, flounder, plaice, pollock, hake	<0.0001	0.2023
Herring, sardine and tuna products	<0.0001	0.5314
Other fish products	<0.0001	0.3007
Dairy products	<0.0001	0.4877
Eggs	<0.0001	0.5989
Meat and meat products	<0.0001	0.4587
Cereals	<0.0001	0.3146
Fats	<0.0001	0.3918

**Table 5:** The analysis of correlation between the declared number of servings of fish and fish products consumed per month and total dietary vitamin D intake from food in the studied group of young Polish women.

Food Group	*p*	R
Salmon, rainbow trout, herring, eel	<0.0001	0.4790
Halibut, mackerel, brook trout, sole, tuna	<0.0001	0.3694
Cod, flounder, plaice, pollock, hake	<0.0001	0.2083
Herring, sardine and tuna products	<0.0001	0.5314
Other fish products	<0.0001	0.3007

**Table 6:** The analysis of influence of predominantly choosing specific fish species on total vitamin D intake from food, conducted based on the analysis of correlation between vitamin D intake from fish and total dietary vitamin D intake from food, and the comparison of vitamin D intake among participants choosing specific fish species or not.

Fish Species	Predominantly Choosing Specific Fish Species	Mean ± SD (µg)	Median (Min–Max) (µg)	Correlation between Vitamin D Intake from Fish and Total Dietary Intake		*p* for Sub-Group Comparison	
				*p*	R	Vitamin D from Fish	Total Dietary Vitamin D
Salmon	Choosing (*n* = 591)	4.21 ± 3.07	3.31 * (0.52–24.52)	<0.0001	0.6839	<0.0001	<0.0001
	Not choosing (*n* = 441)	3.37 ± 2.73	2.65 * (0.00–19.94)	<0.0001	0.6305		
Rainbow trout	Choosing (*n* = 183)	4.65 ± 3.26	3.59 * (0.52–19.37)	<0.0001	0.7157	<0.0001	<0.0001
	Not choosing (*n* = 849)	3.68 ± 2.86	2.90 * (0.00–24.52)	<0.0001	0.6603		
Herring	Choosing (*n* = 493)	4.56 ± 3.18	3.76 * (0.12–24.52)	<0.0001	0.6791	<0.0001	<0.0001
	Not choosing (*n* = 539)	3.20 ± 2.57	2.55 * (0.00–16.06)	<0.0001	0.5870		
Eel	Choosing (*n* = 8)	4.36 ± 4.37	3.14 * (1.47–14.81)	0.6514	−0.1905	0.1733	0.9416
	Not choosing (*n* = 1024)	3.85 ± 2.94	3.09 * (0.00–24.52)	<0.0001	0.6821		

* Non-parametric distribution (verified using Shapiro–Wilk test, *p* ≤ 0.05).

**Table 7:** The results of the logistic regression for the association between predominantly choosing specific fish species and meeting the recommended vitamin D intake for the levels of 5 µg and 10 µg.

Predominantly Choosing Specific Fish Species as a Predictor		Odds Ratio	95% Confidence Interval	Wald Stat	*p*-Value
Model developed for the level of 5 µg	Salmon	1.5793	1.1478–2.1730	7.7413	0.0050
	Rainbow trout	1.5851	1.0942–2.2962	5.8315	0.0148
	Herring	2.5356	1.8544–3.4670	33.3889	<0.0001
	Eel	0.4797	0.0580–3.9655	0.4567	0.4954
Model developed for the level of 10 µg	Salmon	1.3765	0.7495–2.5278	1.0615	0.3029
	Rainbow trout	1.6671	0.8599–3.2317	2.2898	0.1302
	Herring	1.9413	1.0696–3.5235	4.7570	0.0292
	Eel	2.8019	0.3307–23.7382	0.8931	0.3446
